# Using highly variable warfarin dosing to identify patients at risk for adverse events

**DOI:** 10.1186/1477-9560-9-14

**Published:** 2011-10-10

**Authors:** Lyndonna Marrast, Mary Evans, Al Ozonoff, Lori E Henault, Adam J Rose

**Affiliations:** 1 Department of Medicine, Boston University School of Medicine, Boston Medical Center, Boston, MA, USA; 2 Biostatistics Section, Children's Hospital of Boston, Boston, MA, USA; 3 Center for Health Quality, Outcomes, and Economic Research, Bedford VA Medical Center, Bedford, MA, USA

**Keywords:** anticoagulants, dose variability, medication therapy management, risk factors, warfarin.

## Abstract

**Background:**

Patients who receive highly variable doses of warfarin may be at risk for poor anticoagulation control and adverse events. However, we lack a system to identify patients with the highest dose variability. Our objectives were to develop a scoring system to identify patients with high dose variability, and to validate this new measure by demonstrating that patients so identified have poor anticoagulation control and higher rates of adverse events (criterion validity).

**Methods:**

We used a database of over 4, 000 patients who received oral anticoagulation in community practice between 2000-2002. We reviewed the charts of 168 patients with large warfarin dose variation and agreed on 18 risk factor definitions for high dose variability. We identified 109 patients with the highest dose variability (cases), as measured by coefficient of variation (CoV, SD/mean). We matched each case to two controls with low dose variability. Then, we examined all 327 charts, blinded to case/control status, to identify the presence or absence of the 18 risk factors for dose variability. We performed a multivariable analysis to identify independent predictors of high CoV. We also compared anticoagulation control, as measured by percent time in therapeutic range (TTR), and rates of adverse events between groups.

**Results:**

CoV corresponded with other measures of anticoagulation control. TTR was 53% among cases and 79% among controls (p < 0.001). CoV also predicted adverse events. Six cases experienced a major hemorrhage versus 1 control (p < 0.001) and 3 cases had a thromboembolic event versus 0 control patients (p = 0.04). Independent predictors of high dose variability included hospitalization (OR = 21.3), decreased oral intake (OR = 12.2), use of systemic steroids (OR = 6.1), acetaminophen (OR = 4.0) and antibiotics (OR = 2.7; p < 0.05 for all).

**Conclusion:**

CoV can be used to identify patients at risk for poor anticoagulation control and adverse events. This new measure has the potential to identify patients at high risk before they suffer adverse events.

## Background

Warfarin is the standard anticoagulation treatment for atrial fibrillation, venous thromboembolism (VTE), and mechanical heart valves [1-4]. Close monitoring of the International Normalized Ratio (INR) is required due to the drug's very narrow therapeutic window. Many factors can affect INR levels [1,5,6]. Values must be kept within range to reduce the risk of hemorrhage [7,8] and the risk of developing thromboembolism [9]. Previous studies have shown that patients experiencing better anticoagulation control have fewer such adverse events [10-14].

Assessment of adequate anticoagulation control has traditionally been determined by examining INR values themselves, through summary statistics such as percent time in therapeutic range (TTR) [15] or INR variability [16,17]. Several studies have explored the patient-level predictors of control as measured by TTR [10,18,19]. However, there is reason to believe that variability in warfarin doses could also serve to identify patients who are experiencing poorly controlled anticoagulation, thus placing them at risk for adverse events.

We therefore used a large, nationally representative database of community-based oral anticoagulation care to address three related questions. First, we sought to develop a measure of warfarin dose variability that could be used to describe a population and identify patients with highly variable doses over time. Second, we sought to internally validate this new dose variability score as a measure of anticoagulation control using criterion validity. That is, we sought to demonstrate that patients identified as having high dose variability have worse anticoagulation control as measured by TTR and are at higher risk for adverse events than patients with less variability. Finally, through chart review, we sought to identify patient-level predictors of high dose variability. Our overarching goal was to develop a score that could be used to identify patients at high risk for complications.

## Methods

### Database

The Anticoagulation Consortium to Improve Outcomes Nationally (ACTION) study was a large prospective cohort study designed to assess the management of warfarin in community practice within the US [19-21]. A total of 101 participating sites in 31 states recruited 6761 patients receiving long-term oral anticoagulation. All sites used a freely-available software package called CoumaCare for tasks such as patient tracking and recording clinical data. In the database, clinicians updated patient's weekly warfarin dose at each visit. Because the present study relied upon chart reviews, we limited this study to the 47 sites of care that recorded complete notes for at least 90% of INR values. Excluded sites recorded notes only when the INR was not therapeutic. Therefore, this study was limited to 4489 patients.

Enrollment in ACTION occurred between April 2000 and February 2002. Patients were eligible to participate if they were 18 years or older and able to provide informed consent. All data were collected and their completeness rigorously ensured by McKesson HBOC, an independent data management organization. Missing data fields and data entry errors were resolved directly with the sites by the data coordinating center on a weekly basis before the data were transmitted to study investigators. The study protocol was approved by the Western Institutional Review Board of Olympia, WA, and by local review boards where they existed.

Patients were eligible for inclusion in the present study if they had an INR target range of 2-3. Indications for anticoagulation were grouped as follows: atrial fibrillation, venous thromboembolism, valvular heart disease/prosthetic heart valve, and all others. The database included demographics (age, gender, and race) and several comorbid conditions (coronary artery disease, congestive heart failure, hypertension, diabetes) as recorded by the patients' clinicians. Weekly dose of warfarin was recorded for all patients in the database, and was updated by clinicians at each visit. We used these weekly doses to assess the stability of warfarin dose over time for each patient, as will be explained below.

### Chart Reviews

We performed two separate chart reviews, in our efforts to create a score that describes patients with high warfarin dose variability. The first review was implicit; it was performed by chart reviewers without relying upon pre-established definitions. Three physician examiners (LM, ME, and AJR) independently reviewed the charts of 168 patients who had a 2-fold or greater difference between the lowest and highest weekly warfarin dose (e.g. 14 mg/week versus 28 mg/week). The concept behind the review was to remain open to the possibilities of factors that may be present in the database rather than rely solely upon preconceived ideas. Next, the reviewers met and compiled a list of 18 variables believed to have played the greatest role in the dose variability. They reached a consensus regarding a standard definition for each variable in the chart review instrument (Table 1).

**Table 1:** Chart Review Instrument

	Variable	Definition
1	Diet	Any mention of "greens", specific foods high in vitamin K, and dietary content of vitamin K. DOES NOT INCLUDE statements that the vitamin K content of the diet is unchanged.
2	Dietary Supplements	Any mention of multivitamins, Ensure, Boost, Slimfast, etc. as they relate to vitamin K intake. DOES NOT INCLUDE simply listing a multivitamin in the medication list.
3	Adherence	Any mention of problems with adherence to pill-taking, including unauthorized self-adjustment of doses and memory issues. DOES NOT INCLUDE dose confusion after a hospital stay and DOES NOT INCLUDE aspects of adherence (diet, lab follow up, etc.) beyond pill-taking.
4	Hospital or Nursing Home Stay	Any mention of a hospital or nursing home stay EXCEPT for CHF (because that has its own variable - see below)
5	Nausea and Vomiting	Any mention
6	Decreased PO Intake or Decreased Appetite	Any mention
7	Diarrhea	Any mention
8	Decompensated CHF	Any mention of fluid overload, fluid retention, edema, pulmonary edema. Any titration of lasix doses, trending of weight regarding fluid status, use of metolazone (i.e. zaroxolyn), or any obvious CHF regimen. Any hospital admissions for fluid overload.
9	Alcohol	Any mention of alcohol except "denies." Exception - one serving per day or less does not count
10	Amiodarone	Any mention of amiodarone or its brand names "pacerone" or "cordarone."
11	Acetaminophen	Any mention of acetaminophen, products containing acetaminophen. Includes the abbreviation "APAP."
12	NSAIDS/COX-2 Inhibitors	Any mention at all, including mention in the medication list.
13	Procedures	Any mention of a procedure in conjunction with a dose reduction or a "hold" of warfarin - even if the procedure is ultimately cancelled.
14	Cancer	Any mention of cancer, with or without specific therapies such as chemotherapy, radiation, etc. DOES NOT INCLUDE a mere history of cancer.
15	Missed Appointments	Any recorded missed appointments - unless due to hospitalization (which is a different variable).
16	Systemic Corticosteroids	Any mention. DOES NOT INCLUDE joint injections, skin creams, etc.
17	Alternative Medications	Any mention - including but not limited to saw palmetto, St. John's Wort, Echinacea, Coenzyme Q10, etc.
18	Antibacterial Antibiotics	Any mention - must be systemic therapy, not local (such as skin creams, etc.)

For all items, one mention is sufficient to mark the item "yes." Mark a "1" if present, or a "0" if absent.

We found that the criterion used to identify patients with high dose variability (i.e. twofold or greater dose range) did not capture the dose variability we had in mind. Specifically, the method identified a relatively large proportion of patients with one or two outlier doses but otherwise stable dosing. Not all of the patients identified by this score seemed to be experiencing the highly variable anticoagulation control that we were trying to capture. We therefore decided to use the coefficient of variation (CoV) to characterize warfarin dose variability. CoV is defined as the standard deviation of the weekly warfarin dose divided by the mean weekly warfarin dose.

We labeled all patients with CoV greater than 0.2 as patients with high dose variation ("cases"). There were 123 such patients, representing 2.7% of the dataset. Patients with CoV below 0.05 (1019 patients, representing 23% of the dataset) were eligible to be controls. Each case was matched to 2 controls within the same site of care. Charts were excluded if: 1) there were no controls available to match the case patients or 2) the patient was new to warfarin (less than 1 month experience as of study entry). A total of 14 cases and 12 potential controls were removed for these reasons, leaving 109 cases and 218 controls.

The reviewers then independently reviewed charts to identify the 18 variables defined in the chart review instrument. This second review was explicit in that it relied upon the variable definitions described in the instrument. During this second chart review, reviewers were blinded to whether the patient was a case or a control patient. If a factor was present at any time, we recorded this indicator as "1" (present) versus "0" (not present). Each reviewer abstracted one-third of the charts. Fifty of the charts were reviewed by all three reviewers to assess inter-rater agreement.

### Adverse Events

Ischemic stroke/systemic arterial embolism, VTE and major hemorrhage were the adverse outcomes of interest. We defined major hemorrhage according to the definition of the International Society of Thrombosis and Haemostasis: a fatal event, an event requiring hospitalization with transfusion of at least two units of packed red blood cells, or bleeding involving a critical anatomical site such as the cranium or the retroperitoneum [22]. All patient progress notes were individually reviewed for evidence of adverse events; events were validated directly with the sites by McKesson.

### Statistical Analyses

Kappa (κ) statistics were computed to assess inter-rater reliability for the second chart review. To assess significance of effects when comparing categorical variables with the matched design, we used Monte Carlo permutation methods with 10, 000 iterations to compute empirical p-values. Case-control status within each "cluster" of matched observations was randomly permuted 10, 000 times, with a test statistic (e.g., Pearson's chi-square statistic) calculated upon each iteration. This was used as a reference distribution, under the null hypothesis of no association with case status, to compute the empirical p-value. Groups were compared on continuous variables using a generalized linear model to account for correlation between each case and its matched controls. We used conditional logistic regression models to determine the factors that independently predict case status while controlling for patient level covariates (i.e. age, gender, race, co-morbid conditions). Analyses were performed using SAS, version 9.1 (SAS Corporation) and R, version 2.8 (R Foundation).

## Results

### Baseline Characteristics

There were 109 cases with high dose variability and 218 site-matched controls with low dose variability (Table 2). The mean coefficient of variation (CoV) of the cases was 0.24 and the mean for the controls was 0.02 (p < 0.001). The two groups were similar in demographics: most participants were white (89% of cases and 94% of controls) and many were 75 years of age or older (50% of cases and 42% of controls). Forty-five percent of cases were female, compared to 32% of controls (p = 0.02). Atrial fibrillation was the indication for anticoagulation in 67% and 58% of the cases and controls, respectively. Among the 69 patients with "other" indications for anticoagulation, 27 were anticoagulated for stroke, transient ischemic attack, or cerebrovascular disease; 22 were anticoagulated for congestive heart failure; 13 were anticoagulated for coronary artery disease; 4 were anticoagulated for hypercoagulability; and 3 were anticoagulated for other reasons. Co-morbidities such as hypertension, diabetes, and coronary artery disease were similar between groups.

**Table 2:** Baseline patient characteristics compared between cases (n = 109) and controls (n = 218).

Demographics	Cases (%)	Controls (%)	P-value
Age 75 or Older	50	42	0.20*
Female	45	32	0.02*
Nonwhite Race	11	6	0.10*
Hypertension	48	47	0.99*
Diabetes	21	21	0.99*
Coronary Artery Disease	39	34	0.53*
Follow up time	10.9 months	11.5 months	< 0.001†
# INR/month	2.3	1.2	< 0.001†
Indication:			0.26*
Atrial Fibrillation	67	58	
VTE	11	13	
Valvular Heart disease	6	5	
Other	16	24	

*Comparison via Monte Carlo simulation

†Comparison via Generalized Estimating Equations (GEE)

### Validation of Coefficient of Variation as a Measure of Risk

CoV corresponded well with other measures of anticoagulation control and risk for adverse events. The 109 case patients had a mean TTR of 53%, compared to 79% for the 218 control patients (p < 0.001). Cases had a higher rate of adverse events. Six case patients experienced major hemorrhage, compared to only 1 control patient (p < 0.001). Three case patients had thromboembolic events (2 embolic strokes and 1 pulmonary embolism), compared to 0 control patients (p = 0.04).

### Predictors of Dose Variability

We assessed predictors of dose variability using chart review. The 3 reviewers achieved a very good rate of inter-rater reliability (three-way κ = 0.76). In the unadjusted analysis (Table 3), most of the risk factors we examined were associated with case status. Particularly strong associations were seen with amiodarone (12 cases vs. no controls, p < 0.001) and a diagnosis of cancer (8 cases vs. 1 control, p < 0.001). When present, these variables were highly indicative of high CoV.

**Table 3:** Proportion of cases and controls with risk factors for extreme dose variability (unadjusted results).

Risk Factors	Cases (n = 109)	Controls (n = 218)	p-value*
**Acetaminophen**	**33 (30%)**	**30 (14%)**	**< 0.001**
**Adherence**	**48 (44%)**	**67 (31%)**	**0.01**
Alcohol	9 (8%)	16 (7%)	0.99
Alternative Medication	11 (10%)	10 (5%)	0.08
**Amiodarone**	**12 (11%)**	**0 (0%)**	**< 0.001**
**Antibiotic Use**	**47 (43%)**	**44 (20%)**	**< 0.001**
**Cancer**	**8 (7%)**	**1 (0%)**	**< 0.001**
**CHF (Decompensated)**	**14 (13%)**	**7 (3%)**	**0.001**
**Decreased Oral Intake**	**21 (19%)**	**8 (4%)**	**< 0.001**
**Diarrhea**	**15 (14%)**	**10 (5%)**	**0.003**
Dietary Supplement	8 (7%)	6 (3%)	0.06
Dietary Vitamin K	40 (37%)	73 (34%)	0.61
**Hospitalizations/Nursing Home**	**47 (43%)**	**12 (6%)**	**< 0.001**
Missed Appointments	12 (11%)	22 (10%)	0.99
**Nausea/Vomiting**	**10 (9%)**	**5 (2%)**	**0.01**
**NSAID Use**	**19 (17%)**	**18 (8%)**	**0.02**
Procedures	28 (26%)	41 (19%)	0.21
**Systemic Steroids**	**12 (11%)**	**9 (4%)**	**0.03**

All variables were obtained by chart review and all are yes/no variables. Boldface variables are significant at the 0.05 level.

*Via Monte Carlo simulation

After adjustment for covariates (Table 4), variables independently associated with large dose variation included hospital/nursing home stay (OR = 21.3), decreased oral intake (OR = 12.2), use of systemic steroids (OR = 6.1), use of acetaminophen (OR = 4.0), and use of antibiotics (OR = 2.7). Effect size of amiodarone and cancer could not be calculated because there were too few controls with these variables. The presence of these variables precluded model convergence; therefore, these variables were omitted from the model.

**Table 4:** Multivariate analysis of risk factors for extreme warfarin variability.

Chart Review Variables	Odds Ratio (95% CI)	p-value
**Acetaminophen**	**4.0 (1.33 to 6.30)**	**0.01**
Adherence	2.0 (0.87 to 4.65)	0.10
Alcohol	2.5 (0.65 to 10.00)	0.18
Alternative Medication	2.0 (0.38 to 9.63)	0.44
Amiodarone	*	*
**Antibiotic Use**	**2.7 (1.11 to 6.33)**	**0.03**
Cancer	*	*
CHF (Decompensated)	2.0 (0.34 to 11.58)	0.44
**Decreased Oral Intake**	**12.2 (2.25 to 65.68)**	**0.004**
Diarrhea	2.8 (0.51 to 15.67)	0.23
Dietary Supplement	1.0 (0.12 to 7.90)	0.98
Dietary Vitamin K	2.1 (0.86 to 4.92)	0.10
**Hospitalizations/Nursing Home**	**21.3 (6.21 to 73.14)**	**< 0.001**
Missed Appointments	1.6 (0.51 to 5.15)	0.42
Nausea/Vomiting	4.4 (0.70 to 27.91)	0.11
NSAID Use	1.3 (0.36 to 4.72)	0.69
Procedures	1.4 (0.59 to 3.36)	0.44
**Systemic Steroids**	**6.1 (1.10 to 34.20)**	**0.04**

Variables are adjusted for all other variables in the table, as well as for age, gender, race, and comorbid conditions (not shown).

* These variables were not estimable in the multivariate model, because too few control patients had these characteristics. Therefore, these variables were omitted from the model.

## Discussion

In this study, we have describe a new measure to identify patients at risk for adverse outcomes of anticoagulation care, have shown that the measure is correlated with INR control and adverse events, and have examined patient-level predictors of being in this high-risk group. The characteristics independently predictive of large weekly variation in warfarin dose were hospitalization/nursing home stay, decreased oral intake, use of systemic steroids, acetaminophen, and antibiotics. In addition, the use of amiodarone and a diagnosis of cancer were almost certainly risk factors for high CoV, though we could not estimate an effect size.

This study suggests that CoV could be an important tool for identifying patients at high risk for poorly controlled anticoagulation therapy and adverse events. Patients identified as high-risk might be referred for case management, adherence training, more intensive follow-up, or indeed reconsideration of whether this particular patient is a good candidate for warfarin. The utility of such an approach for preventing adverse events could be examined in a prospective study. Anticoagulation control (as measured by TTR) could also be used to prospectively identify patients at high risk for adverse events. Our study did not directly compare the ability of these two measures (TTR vs. dose CoV) to identify patients at highest risk for adverse events; this would also be a suitable topic for future study. We suspect that, in many care settings, there is no effort to prospectively identify patients at high risk of adverse events. If the utility of this approach can be established, it may be more widely employed.

An ideal next step to further this research would be to use CoV to identify patients at high risk for poor outcomes in the context of a quasi-experimental design. At some sites of care, patients with extremely high CoV might be referred for case management, adherence training, more intensive follow-up, or indeed reconsideration of whether this particular patient is a good candidate for warfarin. At other sites of care, CoV would be noted, but not acted upon. The outcomes for patients with high CoV (TTR and hopefully clinical outcomes) would be compared, and the effectiveness and cost-effectiveness of the intervention assessed.

Hospitalization had the strongest association with unstable anticoagulation control of any variable in our multivariate analysis. Being hospitalized can contribute to variable dosing for several reasons. When patients are hospitalized, warfarin therapy is often interrupted, and patients may receive parenteral anticoagulation or no anticoagulation at all. Hospitalization also involves large changes in the patient's lifestyle and diet. Returning home, the patient attempts to re-establish usual habits while often restarting warfarin therapy at the previous dose. Unsurprisingly, this combination of circumstances produces out-of-range INR values. Hospitalization is also a general marker of illness severity, which can predict poorer anticoagulation control both before and after hospitalization. Previous studies have also examined the event of a hospitalization as a time-dependent inducer of variable anticoagulation control [23].

Several studies have shown an association with warfarin and acetaminophen [24,25]. Hylek et al. [26] described acetaminophen as an underrecognized source of INR elevation. Her study which included a case-control prospective design assessed patients with high INR values (> 6.0). Acetaminophen was noted as a risk factor that was documented only as case studies in the literature previously. One study examined the prevalence of adverse warfarin-drug combinations in a post-mortem toxicology database. Acetaminophen accounted for more than half of the warfarin drug interactions. In that study, there were more deaths with the combination of acetaminophen and warfarin than with either drug alone [27]. Despite these data, discordant findings showing lack of an association with acetaminophen and warfarin potentiation have been reported [28-30]. The present study reinforces the theory that the use of acetaminophen can contribute to poor anticoagulation control.

Several other studies have described factors associated with anticoagulation control [31-33]. One study, similar to ours, examined factors that contribute to unstable control and found no association with dietary habits or the presence of comorbid conditions. Instead, they found greater instability among patients working full-time, among those with inadequate understanding of oral anticoagulation therapy, and among those with CYP2c9*3 variants [31]. Other studies have examined factors associated with extremely stable control. Witt et al. [32,33] performed 2 studies looking at patients that spent 100% of the time in therapeutic range. Both studies found that older age, lack of co-morbidities and a standard INR target range (i.e. 2-3) were associated with stable control.

There are several strengths to our study. We used a large, nationally representative database of patients receiving warfarin in community-based practice. Our three chart reviewers achieved a very good rate of inter-rater reliability. Finally, this database (ACTION) contains weekly warfarin doses for all patients. These data are usually not available, since warfarin is often prescribed "use as directed" and so dose changes cannot be reliably abstracted. This is a unique feature of this database, without which we could not have performed this study.

Despite this our study has some limitations. First, this study did not address the question of whether high dose variability is a cause or a consequence of poor anticoagulation control, although we would suspect that it is predominantly a consequence of it. Nevertheless, this study does demonstrate that dose variability is both measurable and related to important clinical outcomes, regardless of its causal relationship with anticoagulation control. As such, it might be used to identify patients at elevated risk for adverse events. Second, this study was limited to risk factors for high CoV that were clearly documented in the clinical notes; however, some risk factors may have been present, but poorly recognized or poorly documented. Our results with regard to risk factors for high CoV should be regarded as exploratory, particularly where a risk factor was shown not to predict high CoV, because an absence of documentation is not conclusive proof that something did not occur. Third, we emphasize that we have only subjected our new scoring system to internal validation, i.e. within the same dataset. A higher level of validation would be attained by demonstrating its utility in a separate dataset. Fourth, the confidence intervals identified in our multivariable analysis of patient-level risk factors for high dose variability are quite large. Therefore, the true magnitude of these effects is not precisely known. A final limitation is that this study evaluates patients with a target INR range of 2-3 and at least 1 month of experience with warfarin; our study results may not apply to patients who are new to warfarin or those with other target ranges.

## Conclusions

In this study, we have derived and internally validated a new measure to identify patients at high risk for poor anticoagulation control in clinical practice, namely the coefficient of variation of weekly warfarin doses. This measure identifies patients at high risk for poor anticoagulation control and adverse events. Future studies should explore the use of this measure to identify patients for intervention before they have experienced an adverse event.

## List of Abbreviations

VTE: Venous Thromboembolism; INR: International Normalized Ratio; TTR: Percent Time in Therapeutic Range; ACTION: The Anticoagulation Consortium to Improve Outcomes Nationally; CoV: Coefficient of Variation; OR: Odds Ratio.

## Competing interests

Data collection for this study was sponsored by Bristol-Myers Squibb. The sponsor was not involved in the study design, study management, data collection, analysis, writing, revision, or decision to submit for publication. The authors do not have any other conflicts of interest to report.

## Authors' contributions

LM helped conceive the study idea, performed chart reviews, and drafted the manuscript. ME helped conceive the study idea and performed chart reviews. AO performed statistical analyses and provided statistical supervision. LEH helped collect the data, performed statistical analyses, and managed the data. AJR helped conceive the study idea, performed chart reviews, performed statistical analyses, and provided study supervision. All authors participated in interpretation of interim results, made revisions to the manuscript for important intellectual content, and approved the final manuscript.

## Authors' Information

LM and ME were third year internal medicine residents at Boston Medical Center at the time this study was performed. The results of this study were presented at the Society of General Internal Medicine's 33^rd ^annual conference in Minneapolis, MN on April 30, 2010.
